# An Acetylcholine Receptor Keeps Muscles in Balance

**DOI:** 10.1371/journal.pbio.1000268

**Published:** 2009-12-22

**Authors:** Rachel Jones

**Affiliations:** Freelance Science Writer and Editor, Welwyn, Hertfordshire, United Kingdom

**Figure:**
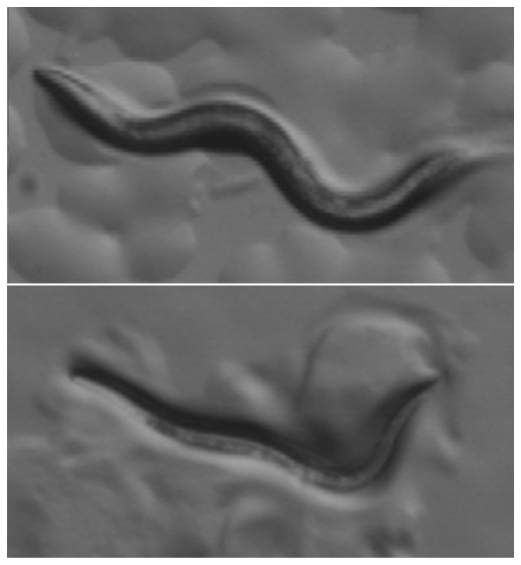
A five-subunit acetylcholine channel coordinates muscle contraction and relaxation by regulating motor neuron excitability in *C. elegans*, allowing the worm to move in a sinusoidal manner (top panel). A mutation in the pore-lining domain of the ACR-2 subunit causes muscle hyper-contraction, due to imbalanced inputs from the motor neurons (bottom panel). (The worm's head is to the left.) (Image Credit: Tamara M. Stawicki and Yishi Jin).

Muscle contraction is controlled by receptors in the muscle cell membranes that respond to the neurotransmitter acetylcholine when it is released from motor neurons. Acetylcholine receptors are also found on neurons, where they perform a variety of important functions, including modulating cognition and addiction. In a new study in *PLoS Biology*, Yishi Jin and colleagues have identified and characterized a neuronal acetylcholine receptor in the *Caenorhabditis elegans* that allows the tiny worm to wriggle about. The receptor regulates the balance between excitation and inhibition in muscles, and thus contributes to the coordinated contraction and relaxation of muscles on opposite sides of the body that results in locomotion.

An acetylcholine receptor consists of five subunits, and there are many subunit types (29 in *C. elegans*) from which a receptor can be assembled. The subunit composition of a receptor, and, in particular, of its transmembrane pore, which is lined with one transmembrane domain from each subunit, determines how it responds to acetylcholine and what effects this response has on the cell. Because of the number of possible subunit combinations, it is very difficult to identify the cell-specific composition of an acetylcholine receptor.

In this study, the authors began by identifying a mutant strain of *C. elegans* in which the muscles were overstimulated, causing the worms to “shrink”, as all their muscles contracted when they were touched. Molecular characterization of the mutation revealed that it consisted of an activating mutation in an acetylcholine receptor subunit called ACR-2. Specifically, the mutation is in the pore-forming transmembrane domain, in a position that is thought to influence the ion selectivity of the channel.

When the authors used reporter genes in which fluorescent proteins were controlled by the *acr-2* promoter, they discovered that the ACR-2 subunit is expressed in cholinergic motor neurons in the worm's ventral cord. Expression of wild-type *acr-2* or a “mini-gene” containing part of the gene in the mutant worms reversed the “shrinking” defect, confirming that the defect resulted from the *acr-2* mutation. Worms in which *acr-2* contained a loss-of-function or null mutation do not show hypercontraction of muscles, but rather moved slowly, and electrophysiological analysis showed that the release of acetylcholine from the motor neurons was reduced in these animals.

In worms with the activating mutation of *acr-2*, the release of acetylcholine from motor neurons was increased. In addition, neurotransmission from inhibitory GABA-releasing motor neurons was reduced. However, ACR-2 is not found in GABAergic neurons, so this reduction in GABA neurotransmission is likely to be an indirect result of the mutation's effects on cholinergic neurons.

To find out which other subunits combine with ACR-2 to make a functional receptor on cholinergic motor neurons, the authors looked for mutations in other genes that suppressed the effects of the activating *acr-2* mutation. Several such mutations were found, and most of these mutations mapped to three other acetylcholine receptor subunit genes—*acr-12*, *unc-38*, and *unc-63*. Other suppressor mutations mapped to genes that are required for transport of the acetylcholine receptor to the cell surface.

To confirm the subunit composition of the acetylcholine receptor, the authors reconstituted the receptor in *Xenopus* oocytes and discovered that in addition to the ACR-2, ACR-12, UNC-38, and UNC-63 subunits, the functional receptor also required ACR-3. The *acr-*3 gene is very close to *acr-2*, so that the two subunits are likely to be coexpressed.

Together, these results show that the ACR-2 neuronal acetylcholine receptor manages the interplay between excitation and inhibition in the muscles in *C. elegans*. They also demonstrate that a gain-of-function mutation in the pore-forming domain of a receptor subunit can influence the pharmacological function of the receptor channel so that transmitter release from the receptor-bearing neuron is increased. Finally, the authors show how the analysis of suppressor mutations can be used to address the challenge of defining the subunit composition of a heteromeric receptor.

Further study of how ACR-2 modulates the excitation and inhibition of muscles might give insight into how this balance is maintained in other neuronal contexts, and how it can be perturbed, for example in some forms of epilepsy. It will be particularly interesting to investigate how an activating mutation in a receptor on a cholinergic neuron can influence the activity of GABAergic neurons that do not bear the mutated channel.

**Jospin M, Qi YB, Stawicki TM, Boulin T, Schuske KR, et al. (2009) A Neuronal Acetylcholine Receptor Regulates the Balance of Muscle Excitation and Inhibition in** ***C. elegans*** **. doi:10.1371/journal.pbio.1000265**

